# Cathepsin L and acute ischemic stroke: A mini-review

**DOI:** 10.3389/fstro.2022.1050536

**Published:** 2022-11-10

**Authors:** Linda Ma, Silin Wu, Aaron M. Gusdon, Hua Chen, Heng Hu, Atzhiry S. Paz, Jaroslaw Aronowski, Jude P. Savarraj, Ryan S. Kitagawa, Huimahn A. Choi, Xuefang S. Ren

**Affiliations:** ^1^Department of Neuroscience, Center for Basic and Translational Stroke Research, Rockefeller Neuroscience Institute, West Virginia University, Morgantown, WV, United States; ^2^Division of Neurocritical Care, Department of Neurosurgery, McGovern School of Medicine, University of Texas Health Science Center, Houston, TX, United States; ^3^Department of Neurology, McGovern School of Medicine, University of Texas Health Science Center, Houston, TX, United States

**Keywords:** cathepsin L, acute ischemic stroke, extracellular matrix, middle cerebral artery occlusion (MCAO), metalloproteinase

## Abstract

Ischemic stroke is a serious cerebrovascular event that results in cell death, blood-brain barrier dysfunction, tissue degradation, and inflammation, often leading to permanent disability or death. As the incidence of ischemic stroke continues to rise globally, it is crucial to examine the mechanisms of the various proteins and molecules contributing to worsened patient outcome and recovery. Cathepsin L, a cysteine protease known for degrading tissues in lysosomes and elsewhere, may play a role in brain tissue loss and inflammation after stroke. Studies have suggested that cathepsin L appears in the ischemic core shortly after stroke is induced. Using immunohistochemical staining, mass spectrometry, and other assays, the increase of cathepsin L in the brain was correlated with extracellular matrix and perlecan degradation after ischemic stroke. Additionally, injection of a cathepsin L inhibitor significantly reduced brain infarct size and improved functional scores. More research is needed to elucidate cathepsin L's role in post-stroke inflammation and brain damage, in order to further explore the factors contributing to worsened patient outcome after ischemic stroke and work toward finding better therapeutic interventions.

## Introduction

Stroke is a leading cause of cerebrovascular accidents (CVAs), affecting over 33 million people a year globally (Feigin et al., 2014). CVAs causes two types of brain injury: ischemic stroke and hemorrhagic stroke. Ischemic stroke involves brain vessel occlusion and blockage and accounting for about 80% of all CVAs (Donnan et al., 2008). Symptoms from ischemic stroke manifest slowly over several hours, depending on the area of the brain affected (Ojaghihaghighi et al., 2017) and a cascade response to an ischemic core. The ischemic cascade, referring to the neurochemical events in the penumbra set in motion by focal cerebral ischemia, involves generation of free radicals and usually results in oxidative stress and cell death, blood-brain barrier dysfunction and loss of microvascular integrity (Brouns and De Deyn, 2009). Pro-inflammatory cytokines and chemokines are secreted, causing leukocyte invasion and accumulation in the brain region (Woodruff et al., 2011) and leading to tissue injury and infarction (Huang et al., 2006). At present, many studies have shown that the inflammatory mediation of leukocytes plays an important role in ischemic stroke, including the discovery of these mediator adhesion molecules such as selectins, integrins, and immunoglobulins, cells from the MCAO model. Factors such as IL-1, IL-6, TNF-a and TGF-b) and chemokines such as CINC and MCP-1, these inflammatory factors are produced immediately after an ischemic attack leading to irreversible cerebral infarction (Wang and Lo, 2003) Brain tissue in ischemic stroke suffers from microvascular leakage and rupture due to oxidative damage to lipid-rich membrane structures in the blood-brain barrier. Appears early after the onset of perfusion injury (Chan, 1994; Asahi et al., 2000).

Acute ischemic stroke is classified into permanent or transient stroke as per cerebral blood flow restoration. The brain is the most sensitive to blood supply reduction out of all the organs; with its high metabolic activity and low level of stored glucose, lack of oxygen causes brain damage to set in quickly (Kalogeris et al., 2012). Permanent damage depends on the degree and duration of oxygen deprivation, and it spreads outward from the ischemic core to the penumbra, where the damage can be stopped or reversed in some cases. Transient ischemia may cause reperfusion injury, occurring when blood flows back into the ischemic core rapidly after oxygen deprivation has occurred. As a result, oxidative stress, platelet activation, leukocyte activation, and other mechanisms cause the functional outcome of stroke to be worsened (Lin et al., 2016). Another effect is a decrease in neuron density, occurring after 24 h of reperfusion when using a primate model (Hamann et al., 1996). A computed tomography CT scan uses a series of X-rays to create detailed images of the brain. CT scans can show brain hemorrhages, ischemic strokes, tumors, or other diseases. Computed Tomography Scanning Angiography is used to see blood vessels in the neck and brain in more detail (Kidwell and Wintermark, 2008). To detect the area of damage in the brain, computed tomography (CT) and magnetic resonance imaging (MRI) scans are generally used. Computed tomography (CT) has been widely used in the diagnosis of brain trauma, stroke, and other diseases due to its rapid detection and ease of use (Kidwell and Wintermark, 2008). However, compared with CT, magnetic resonance imaging (MRI) has unique advantages in accuracy. Though MRI scan duration is longer than CT, it is known to be more sensitive in the detection of ischemia (Vymazal et al., 2012). Magnetic Resonance Imaging (MRI) uses strong radio waves and a magnetic field to create a detailed view of your brain. MRI can be used to detect brain tissue damage from ischemic stroke and cerebral hemorrhage (Thomalla et al., 2018). Magnetic resonance angiography, or magnetic resonance venography, provides a better view of intracranial arteries and veins. For ischemic stroke, the use of diffusion-weighted imaging (DWI) sequences can rapidly detect the area of acute ischemia, thereby providing a reference for patients to select an appropriate endovascular treatment time window as soon as possible (Leslie-Mazwi et al., 2016; Nogueira et al., 2018). Additionally, Carotid ultrasonography uses sound waves to generate detailed images of the inside of the carotid arteries in the neck, showing fatty deposits (plaques) and blood flow in the carotid arteries. Cerebral angiography, as the gold standard for diagnosing cerebrovascular disease, can be seen in detail under X-ray imaging to observe cerebral arteries and carotid arteries (Powers et al., 2018).

Although significant efforts have been made in improving preventive and supportive measures, it is more difficult to develop treatments to combat the effects of ischemic stroke. Intravenous thrombolytic therapy is currently the most important measure to restore blood flow. Recombinant tissue plasminogen activator (rt-PA) and urokinase are currently commonly used thrombolytic drugs. Presently, the only available therapies are thrombolysis with tissue plasmogen activator (tPA) and surgical removal of blood clots (Iadecola and Anrather, 2011). However, most stroke victims only receive supportive care due to safety concerns and a narrow time frame (≤4.5 h after stroke onset) for tPA treatment. In addition, patient, and hospital characteristics such as age, time of day when stroke happened, gender, and hospital inexperience with tPA greatly affected if the treatment was administered in time (Fonarow et al., 2011). The greatest risk of intravenous thrombolysis is hemorrhagic transformation. rt-PA dose and blood pressure were closely related to the risk of hemorrhagic transformation after thrombolysis (Anderson et al., 2016, 2019). Clinically, the benefit and bleeding risk can be assessed based on the specific conditions of the patient, and the dose of rt-PA and the target of blood pressure control can be selected. Endo-vascular interventional therapy is an extremely important surgical intervention, including endovascular mechanical thrombectomy, arterial thrombolysis, and angioplasty. Endovascular mechanical thrombectomy is the most important progress in the treatment of acute ischemic stroke in recent years, which can significantly improve the prognosis of patients with ischemic stroke caused by acute large artery occlusion (Goyal et al., 2017; Kim et al., 2019). Surgery has become an important method for the treatment of hypertensive intracerebral hemorrhage due to its advantages of rapid removal of hematoma, relief of intracranial hypertension, and release of mechanical compression, mainly including craniotomy for hematoma removal, burr hole drainage, decompressive craniectomy, minimal invasive surgery based on precise stereotaxic puncture equipment, ventricular drainage/thrombolytic drug application, etc. (Mendelow et al., 2005, 2013, 2015; Mould et al., 2013).

With very limited treatment options for stroke, improving recovery after cerebral ischemia is crucial; research has been done to study the proteins that may play a role in tissue degradation and damage in the brain after arterial occlusion. Cathepsin L, one of cysteine proteases known for degrading proteins throughout the body, has been linked to such deleterious effects after ischemia. The purpose of this paper is to gather the research on ischemic stroke and cathepsin activity to analyze the relationship between them and the current gaps in knowledge on this topic. As the populations of developed countries age and incidences of stroke become more prevalent, as predicted by epidemiologists using the Dijon Stroke Registry (Bejot et al., 2019), it is ever more important to understand the factors contributing to brain damage after ischemia to search for a possible drug target/treatment to improve recovery.

## Cathepsin L

Many studies have found that human cathepsins are involved in multiple physiological processes and are highly related to most human diseases (Turk et al., 2012; Novinec and Lenarcic, 2013). There are a large number of protease cathepsins in the lumen of the lysosomal structure of almost all organisms, which are a class of endolysosomal proteases based on their active site amino acid residues and different chemical structures. Cathepsins are divided into several types, including serine types: cathepsins A and G, aspartic acid types: cathepsins D and E, and the most numerous cysteine types: cathepsins B, C, F, H, K, L, O, S, V, W and X (Bright et al., 2016; Vidak et al., 2019; Yadati et al., 2020). Cathepsins are widely involved in various cellular activities in cells, such as the synthesis and activation of hormones, the process of apoptosis and autophagy, *etc*. Several studies have found that cathepsins B and L are involved in the pathophysiological process of various neurological diseases, such as ischemic stroke, Alzheimer's disease, Parkinson's disease, and traumatic brain injury (Lipton, 1999; Xu et al., 2013; Yamashima, 2013).

Cathepsin L is a papain-like cysteine proteinase ubiquitously expressed in human tissues and usually active in a slightly acidic pH. Though originally thought to be lysosomal, research has shown that it also is involved in processes in the nucleus, cytoplasm, and plasma membranes of cells (Turk et al., 2012). Besides degrading intracellular and extracellular proteins, cathepsin L also plays a role in regulation of cell-cycle progression (Goulet et al., 2004), as well as being involved in cell signaling and antigen presentation. Cathepsin L was shown to process proneuropeptides into active hormones and neuropeptides in secretory vesicles for cell-cell communication (Funkelstein et al., 2010). Honey et al. (2002) found that mice deficient in cathepsin L showed inefficient selection of CD4^+^ T cells, so cathepsin L may play an important part in the adaptive immune response by generating MHC class II peptide epitopes (Honey et al., 2002). Research has shown that increased levels of cathepsin L are correlated with decreased heart function in dilated cardiomyopathy (Mehra et al., 2017), increased inflammation in rheumatoid arthritis (Weitoft et al., 2015), and increased malignancy of certain cancers (Olson and Joyce, 2015). Since cathepsin L is upregulated in different tumors and can facilitate cancer cell migration, many studies have been conducted to study it as a possible anti-metastasizing drug target (Parker et al., 2015). Cysteine proteases have also been linked to the occurrence of abdominal aortic aneurysms, with aortic wall samples showing significantly higher expression of cathepsin B, H, L and S, supporting the postulation that cathepsins are involved in aneurysm expansion (Abisi et al., 2007).

Our preliminary data have demonstrated that when cerebrovascular epithelial cells from bEND.3 cell line are cultured and subjected to hypoxia ischemia (HI) conditions, cathepsin L activity was decreased in cell lysates and increased in the supernatant (data not shown), indicating that ischemia may activate cathepsin L secretion in endothelial cells.

In addition, research has shown that active cathepsin L released during stroke may play a role in degradation of micro-vessel tissues, worsening stroke outcome. A study conducted by Fukuda et al. using human cathepsin reagents on brain tissue samples from 30 adolescent male baboons examined the immunoreactivity of vascular matrix components. The animals underwent MCAO for 1, 1.5, 2, or 3 hours with varying reperfusion times. It was found that damage to the vascular perlecan after focal ischemia may be due to cathepsins B and L, which appear shortly in the ischemic core after MCAO (Fukuda et al., 2004). This is the first evidence implicating cathepsins B and L in degradation of cerebral tissues.

Cathepsins accumulate lipofuscin leading to rupture of lysosomal membranes. Membrane damage results in the release of cathepsin B, which further stimulates the inflammasome (Cai et al., 2012; Nakanishi, 2020a,b). The formation of lysosomal membranes is dynamic and excessive autophagy will reduce the stability of lysosomal membranes. This process leads to lysosomal membrane leakage, including lysosomal proteases, such as cathepsin B and L, etc. (Canu et al., 2005). In many invasive or metastatic tumors, cysteine cathepsins contain CpG islands, and their promoter regions undergo epigenetic regulatory modifications and affect methylation regulation in tumor cells (Yadati et al., 2020). In addition, cathepsin D is involved in the biological process of endosome trafficking and colocalization in brain neuronal cells (Yamasaki et al., 2007). Deficiency of cathepsin D may delay central nervous system myelination by inhibiting proteolipid protein trafficking from late endosome/lysosome to plasma membrane (Guo et al., 2018). These previous studies suggest that cathepsins might play different roles in early and late stages of stroke. Based on these evidences, cathepsins might exacerbate infarction by stimulating inflammasome during early stages of stroke. However, cathepsins could play a “double-edged sword” role and more evidences are required to conclude their good or bad impact on long-term stroke outcomes.

Gu et al. used cerebral tissue samples from 10 adolescent baboons as well as adult mice. MCAO was achieved in awake animals using an implanted balloon device placed prior to the experimental way to middle cerebral artery occlusion, lasted 3 h. Immunoreactive cathepsin L was found in the ischemic core of both primate and murine models about 2 h after onset of ischemia (Gu et al., 2015). *Ex vivo* protease secretion assays and extracellular matrix degradation assays showed that cathepsin L release during ischemia disturbed micro-vessel integrity. However, limitations of this study included how frozen brain tissue in the experiment can “spill” cathepsin L and other proteinases, which causes unanticipated extracellular matrix degradation.

**Figure 1:**
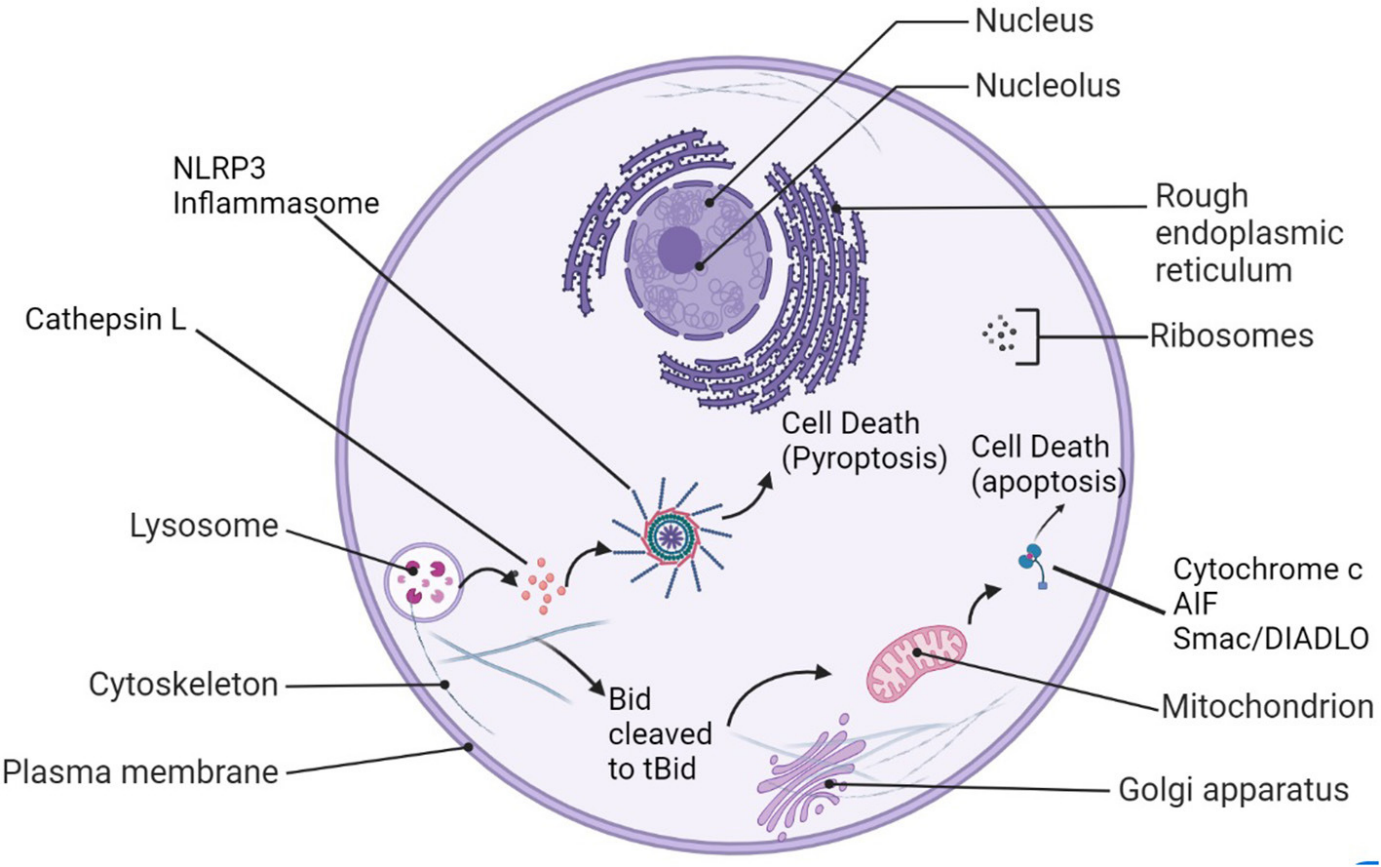
Cathepsin L's roles in cell damage after stroke. After lysosomal rupture/leakage, cathepsin L activates the NLRP3-inflammasome and leads to pyroptosis, or it can cleave Bid to tBid, be translocated to the mitochondrion. Proapoptotic factors such as cytochrome c, AIF, and Smac/Diablo are released into the cytosol as a result and trigger apoptosis.

After stroke, Cathepsin L may indirectly affect autophagy through activation of the cathepsins–tBid–mitochondrial apoptotic signaling pathway (Figure 1) (Papadopoulos and Meyer, 2017). Cathepsins and other lysosomal enzymes induce mitochondrial permeability transition (MPT) by activation of Bid. Bid is converted to tBid, its proapoptotic form, and truncated to mitochondria, where pro-apoptotic factors such as cytochrome c, apoptosis inducing factor (AIF), and Smac/DIABLO are activated and released into the cytosol (Qin et al., 2008). Though generally good for cell survival, autophagy in context of cerebral ischemia is harmful, causing neuronal cell death and apoptosis (Rami and Kogel, 2008). When an autophagy inhibitor 3-MA was injected into male Sprague-Dawley rats after permanent MCAO, cathepsin B and L expression was reduced (Zhou et al., 2017). Though permanent MCAO differs from the transient model used in many other studies to mimic ischemic stroke in humans (the mice show primary core damage, with the maximum damage being achieved at the 3-h mark, unlike tMCAO), the pMCAO model is still widely considered suitable for simulating human ischemic stroke (Fluri et al., 2015).

Cathepsins L and B released into the cytoplasm of cells by lysosomal rupture may also play a role in the activation of the NOD-like receptor protein (NLRP3)-inflammasome (Figure 1), eventually leading to pro-inflammatory cytokine release, exacerbation of tissue injury, and pyroptosis, cell death. There are some researches indicated that Cathepsin L (CatL) is a potent collagenase involved in atherosclerotic vascular remodeling and dysfunction in animals and humans. Studies have suggested that cathepsin L may be involved in the process of apoptosis, and its mechanism is related to the activation of the key apoptotic protein Caspase-3 in neurons (Ishisaka et al., 1999). Genomic studies have found that both Cathepsin L and Cathepsin B are involved in the neuronal death process mediated by microglia β-amyloid (Aβ) activation (Boland and Campbell, 2004). Studies suggest that Cathepsin L is a member of the Aβ-stimulated apoptotic cascade. In addition, pro-cathepsin L secreted outside cells can act on various extracellular matrices and degrade matrix components, thereby affecting cell survival and facilitating tumor cell metastasis (Jean et al., 2006, 2008). Some recent *in vivo* experimental studies have shown that the lysosomal system may be involved in neurodegenerative diseases (Lee et al., 2006; Zhang et al., 2007). Pro-cathepsin L secreted to the outside of cells can act on a variety of extracellular matrices and degrade matrix components, thereby affecting cell survival. In addition, some studies have found that compared with other cathepsin, the protein and enzyme activity of cathepsin L (CTSL) are significantly increased in the carotid artery and blood of aged mice. *In vitro* recombinant CTSL can promote vascular endothelial cells senescence (Chwieralski et al., 2006; Cai et al., 2017; Fu et al., 2017).

## Cathepsin L inhibitors for stroke treatment

These findings above point toward cathepsin inhibition as a possible strategy to reduce micro vessel injury and the other consequences of ischemic stroke.

The selective cathepsin B/L inhibitor Cbz-Phe-Ser(OBzl)-CHN_2_, cysteine protease inhibitor 1 (CP-1), is part of the peptidyl-diazomethane family. It is a small lipophilic polypeptide that can select for cathepsins without also selecting for calpains or caspases. CP-1 was injected into rats immediately following reperfusion after 2 h of MCAO in a study conducted by Seyfried et al. Results showed that infarct volume was significantly reduced in rats given intravenous CP-1 compared to the controls, and CP-1's effect on neurons is thought to be protective instead of directly affecting reperfusion. This is supported by the fact that cerebral blood flow was not affected after injection of CP-1(Seyfried et al., 2001). This data supports that cathepsin inhibition could be beneficial after focal ischemia occurs, by reducing infarct volume post-ischemia. Toxicity has been pointed out as a possible concern about cathepsin inhibitors in cells (Li et al., 2017), but Seyfried et al. noted that the dose used in their experiment did not cause cell toxicity.

Anagli et al. also studied CP-1's effects on postischemic processes with a murine model consisting of male rats subjected to MCAO using the intravascular suture method, discovering that CP-1 caused a significant reduction in serum albumin leakage into the brain, which is a marker of blood-brain barrier integrity. These results were indicative of decreased secondary ischemic damage and improved neurological recovery of the rats used in the study (Anagli et al., 2008), and this supports the idea that cathepsin inhibition may be beneficial for improving ischemic stroke prognosis and recovery.

In 2011, Yang et al. conducted a study on CP-1 using a murine model to examine how injection of the inhibitor affected rats' recovery from induced intracerebral hemorrhage (ICH). Though ICH is not a major part of ischemia, it is thought that the same proteolytic pathways may be activated due to cellular stress. Results showed that the 40 male rats treated with CP-1 had significantly reduced apoptosis and tissue loss compared to controls, as well as increased angiogenesis and synaptophysin density 2 weeks after CP-1 treatment (Yang et al., 2011).

Based on these studies, CP-1 may be helpful in treatment of focal ischemia by improving cellular survival after ICH and lessening neuronal damage. However, more research is needed to fully explore the effects of cathepsin inhibition, the specific mechanisms behind inhibition, and its potential for treating stroke patients. Also, CP-1 was found to be an irreversible inhibitor, which is usually not preferred for drug discovery because irreversible inhibitors permanently change the target protein and may cause an immune response. However, there have been successful drugs created using the same irreversible mechanism (Dana and Pathak, 2020).

The events leading to permanent damage in the brain after ischemic stroke have also been connected to increased metalloproteinase (MMP) activity in plasma, besides cathepsins and other proteases. MMPs are enzymes that have been shown to degrade the extracellular matrix (ECM) of the cerebral basal lamina (Del Zoppo et al., 2012). There are two types of MMPs, constitutive and inducible. The constitutive enzymes, MMP-2 and 14, remain close to the site of activation. The inducible enzymes, MMP-3 and 9, are activated by free radicals and enzymes during inflammation in the brain (Yang and Rosenberg, 2015). Inducible MMP activity has been linked to ischemic reperfusion injury, with myocardial infarct size reduced in MMP-9 knockout mice (Romanic et al., 2002). Heo et al. used gelatin zymography to show that MMPs significantly increased in ischemic basal ganglia of primates along with neuron injury 1 h after MCAO (Heo et al., 1999). Interestingly enough, MMPs have been linked to cathepsins as well; regarding experimental myocardial infarction, it was shown that early cathepsin L release after heart tissue injury correlates with MMP-9 activation (Sun et al., 2011). In another study, when a cathepsin and calpain inhibitor was injected in adult male rats before induced cerebral ischemia, MMP-9 activation was reduced, and infarction volume was decreased overall (Tsubokawa et al., 2006). Ren et al. used the blood replacement treatment for stroke mice and indicated BR therapy possible by reducing the cascading inflammatory response in stroke by decrease the level of MMP-9 in the blood (Ren et al., 2020). Future studies may focus on MMP-9's interactions with cathepsin L and how both might regulate stroke outcomes.

There are cytokines, or small secreted proteins released by cells, that are connected to cathepsin L activity in cancers and other diseases. Pro-inflammatory cytokines often contribute the worsening of conditions such as atherosclerosis and rheumatoid arthritis (Isomaki and Punnonen, 1997). Using bone marrow-derived macrophages from mice, Tumor Necrosis Factor-α and Interleukin-1β were found to be responsible for cathepsin L and S upregulation during the immune response to bacterial lipopolysaccharide (LPS). Interferon-β may also induce cathepsin L secretion, perhaps by a different mechanism (Creasy and Mccoy, 2011). In another study, Interleukin-6 induced upregulation of cathepsin L, whereas Transforming Growth Factor-β1 suppressed cathepsin B and L expression (Gerber et al., 2001). The IL-10-secreting B cells is a major regulatory cell type in stroke, and suggests that the enhancement of regulatory B cells could potentially serve as a new therapy for this devastating neurological disease (Ren et al., 2011). However, it has not been reported if IL-10 modulates stroke outcomes by affecting cathepsin L signal pathway.

The Table 1 below summarizes the research progress on cathepsin L and stroke:

**Table 1:** Summary of research relating cathepsin activity to ischemic stroke.

**References**	**Purpose**	**Sample Size**	**Outcome Measures**	**Findings**
Fukuda et al. (2004)	Examine if active proteases (MMPs, cathepsins) can degrade vascular matrix after MCAO	30 adolescent male baboons	- *Ex vivo* assays quantifying the effects of proteases on the immunoreactivity of vascular matrix components - Protease inhibition tests	- Generation of active MMPs that degraded microvessel tissue - Active cathepsin B and L were generated 2 h after MCAO and were found to degrade perlecan
Gu et al. (2015)	Examine cathepsin L's roles and interactions with MMPs during ischemia	10 adolescent male baboons, unknown number of adult mice	- *Ex vivo* protease secretion assays - Extracellular matrix degradation assays	- Cathepsin L but not B appears in the acidic ischemic core within 2 h - Cathepsin L degrades microvessel matrix and interacts with MMPs
Zhou et al. (2017)	Elucidate the mechanisms of ischemia-induced autophagy using pMCAO and OGD models	Unknown number of male Sprague-Dawley rats	- Immunofluorescence analysis - Western blot analyses - lysosomal staining	- Autophagy inhibition reversed ischemia-induced activation of cathepsin B and L - Inhibition of autophagy blocks cathepsins–tBid–mitochondrial apoptotic signaling pathway
Seyfried et al. (2001)	- Test the selective cathepsin inhibitor CP-1 for cerebroprotectivity after stroke and toxicity - Determine if cathepsins contribute to neuronal death after ischemia	81 male Wistar rats	- *In vitro* toxicity assays - Functional scoring of rat behavior - Examination of rat brain lesion volume	- CP-1 significantly reduced infarct volume and improved rats' functional scores at certain dosages - Lysosomal cathepsins B/L contribute to cerebral injury after ischemia
Anagli et al. (2008)	- Investigate the effects of selective inhibition of cathepsins B/l (using CP-1) on postischemic protein changes	Unknown number of male Wistar rats	- Cathepsin B activity assay - SDS-PAGE and Western Blot Analysis - Mass spectrometry and analysis	- CP-1 reduced infarct volume, neurological deficits, cathepsin B activity, and albumin leakage in the brain - CP-1 helps with neurological recovery after stroke
Yang et al. (2011)	-Investigate CP-1's ability to aid in functional and histological recovery after intracerebral hemorrhage	40 adult male Wistar rats	- Neurological functional studies - Quantified immunohistochemical staining	- Rats treated with CP-1 showed reduced apoptosis/tissue loss - Neurological function improved after CP-1 treatment - Cathepsin B and L are involved in neuronal cell death after ICH
Tsubokawa et al. (2006)	- Investigate the role of the calpain and cathepsin B inhibitor E64d on MMP-9 activation after cerebral ischemia	65 adult male Sprague Dawley rats	- Neurological scoring - Infarction volume evaluation - Morphological assessment and zymography	- Pretreatment with the inhibitor reduced infarction volume and neurological deficits - Cathepsin B and calpain inhibitor E64d inhibits the upregulation of MMP-9 also

## Translational significance

**Figure 2:**
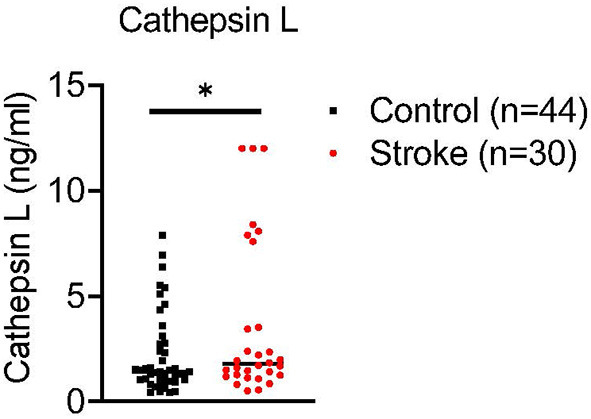
Ischemia increases cathepsin L levels in plasma of acute stroke patients. Blood was collected from combined controls (16 healthy controls and 28 stroke mimic controls) and 30 stroke patients (within 24 hours of stroke onset prior to medical intervention). Cathepsin L levels were significantly elevated in plasma of stroke patients. Mean ± S.D. Student's *t*-test *, *p* < 0.05.

We evaluated levels of cathepsin L in plasma of 30 acute stroke patients (stroke onset within 24 h, confirmed by MRI) compared to age matched controls by enzyme-linked immunosorbent assay. Our preliminary data have demonstrated that cathepsin L is significantly increased in plasma from stroke patients compared to controls (Figure 2). The data suggest that cathepsin L offers a translational significance in stroke. However, it is unclear if cathepsin L is a key culprit that exacerbates stroke evolution, and more research needs to be done to further evaluate the translational value by targeting cathepsin L in stroke.

## Perspectives and conclusion

In summary, the rising number of incidences of ischemic stroke is a public health concern. Though preventive and supportive care has been improved, there is a great need for the development of treatments to combat the cerebral tissue degradation, inflammation, and blood-brain barrier dysfunction occurring after ischemia. Thus, it is prudent to examine the mechanisms of tissue injury to identify possible treatment methods.

The research articles summarized above in this mini-review paper connect cathepsins to many of the harmful after-effects of stroke. Cathepsin L accompanies selective MMPs, serine proteases and other proteases in ischemic regions. Both can indicate changes in the vascular microenvironment caused by focal ischemia. Cathepsins are involved in numerous important processes in cells; in fact, increased expression of cathepsins has been linked to diseases such as dilated cardiomyopathy, cancer malignancy, rheumatoid arthritis, and now focal cerebral ischemia. Many studies have confirmed the potential mechanism of action that microglia activated during ischemia may be damaged by ischemic core area.

Cathepsin L, specifically, appears in the ischemic core in the brain after stroke and is correlated with ECM and perlecan degradation. Studies conducted with CP-1, a selective cathepsin B/L inhibitor, showed that blocking cathepsin expression after MCAO reduced infarct volume and was thought to have a protective effect on neurons. Though CP-1 looks promising as a possible treatment for reducing the effects of ischemia, more research is needed to illuminate cathepsin L's full role in focal cerebral ischemia and the side effects of cathepsin inhibition using the mechanism of irreversible inhibition. Other matrix proteases produced in ischemic stroke also interact with cathepsin L in micro-vessels through activated microglia, leading to matrix destruction under focal ischemic conditions, and this research direction may further establish potential basis for successful treatment of ischemic stroke.

An acute ischemic stroke usually causes systemic pathological changes, and currently, there is no single drug or treatment to eliminate these complicated responses (Brouns and De Deyn, 2009; Ren et al., 2020). Our previous research shows that whole-blood substitution therapy is expected to address the systemic pathological response caused by stroke. Certainly, replacing certain amount of whole-blood in stroke mice may reduce the cascading inflammatory response by decreasing the level of MMP-9 (Ren et al., 2020). This treatment becoming a breakthrough treatment is because this strategy may reduce the mortality of acute stroke patients and improve stroke prognosis, providing a reliable experimental basis for future clinical trials of exchange transfusion therapy (Ren et al., 2020). Although, the role of cathepsin L is not evaluated in this study, replacing whole-blood in stroke mice could reduce cathepsin L level in circulating blood. If cathepsin L is a major culprit of exacerbating infarction following stroke, targeting cathepsin L could effectively improve stroke outcomes rather than a whole-blood replacement therapy. To explore new treatment strategies for stroke, more in-depth studies are required for evidence of how cathepsins regulate the pathological process of stroke.

## Author contributions

XSR conceived and edited the manuscript. LM drafted the manuscript. SW wrote and edited the manuscript. AMG, HC, HH, ASP, JA, JPS, RSK, and HAC performed the research. All authors contributed to the article and approved the submitted version.

## Funding

The study was supported by UTHealth new faculty start-up and NSF funding 1916894 (PI: XSR).

## Conflict of interest

The authors declare that the research was conducted in the absence of any commercial or financial relationships that could be construed as a potential conflict of interest.

## Publisher's note

All claims expressed in this article are solely those of the authors and do not necessarily represent those of their affiliated organizations, or those of the publisher, the editors and the reviewers. Any product that may be evaluated in this article, or claim that may be made by its manufacturer, is not guaranteed or endorsed by the publisher.
